# Income dissatisfaction and migraine headache. Evidence from a nationwide population-based survey

**DOI:** 10.1080/21642850.2023.2266214

**Published:** 2023-10-11

**Authors:** Sandro Rondinella, Damiano B. Silipo

**Affiliations:** a Department of Economics and Statistics, University of Naples Federico II, Napoli, Italy; b Department of Economics, Statistics and Finance ‘Giovanni Anania’, University of Calabria, Rende, Italy

**Keywords:** Income dissatisfaction, Migraine, Comorbidities, Psychological illnesses and migraine, Italy, I1, H51

## Abstract

**Objective:**

We investigate whether and to what extent income dissatisfaction (ID) is an important determinant of migraine. Indeed, ID may play a more relevant role in migraines than realized income, and it may affect both low and high-income people.

**Design:**

We exploit the Italian Statistical Institute (ISTAT) survey covering about 80,000 individuals for this study. On the methodological ground, an instrumental variable probit model has been implemented.

**Main Outcome Measures:**

To measure income dissatisfaction we exploit a self-reported status ranging from 1 to 4, while the migraine variable captures whether the individual suffers from migraine.

**Results:**

The results show that the higher the ID the greater the probability of having a migraine. This relationship is robust to the level of realized income, socioeconomic characteristics of the individual, and the existence of other illnesses.

**Conclusions:**

The high relevance of ID among low-income as well as high-income people opens up a new perspective on the determinants of migraines and provides an explanation of the contrasting evidence in the literature about the income-migraine nexus.

## 1. Introduction

According to the Global Burden of Disease study 2019, migraine is second among the world’s causes of disability, and first among young women (Steiner et al., 2020). And its relevance is increasing over time (Woldeamanuel & Cowan, 2017).

The bulk of the literature points out that the prevalence of migraines is higher among women, people in middle age, and with lower income and socioeconomic status. Other highly related factors are comorbidities, genetic factors, lifestyle and stress conditions. The World Health Organization (2016), Woldeamanuel and Cowan (2017), Jensen and Stovner (2008) reveal that migraine increases with age up to about fifty, and has a decreasing trend thereafter. Almost all the findings show that the percentage of women with migraine is more than double that of men (Bigal et al., 2004; Stang et al., 1992; Stewart et al., 1991; Vetvik & MacGregor, 2017), and differences persist up to 70 years old and beyond (Stewart et al., 1992). There is also widespread evidence that migraine prevalence is higher among people with lower incomes. As an example, Stewart et al. (1992) find that prevalence in the lowest US income group was more than 60% higher than in the two highest income groups. This is confirmed for all other countries and periods studied (Agosti, 2018; Bigal et al., 2004; Fernández-de-Las-Peñas et al., 2010; Molarius et al., 2008; Queiroz et al., 2009; Stewart et al., 2013; Winter et al., 2012). Furthermore, the result holds when we include education as a component of socioeconomic status. Hammond and Stinchcombe (2019) find that perceived social status as well as actual social status is associated with lower migraine. Scher et al. (1999), Warren (2009), Winter et al. (2012), Silberstein et al. (1995), Stewart et al. (2013) find that the higher prevalence of migraine in the lower socioeconomic groups may be a consequence of a circumstance associated with low income, such as poor diet, poor medical care, or stress. Migraine has also been noted to be comorbid with several other illnesses. The most frequent illnesses associated with migraine are depression and anxiety (Agosti, 2018; Buse et al., 2019; Cesur et al., 2015; Korolainen et al., 2019; Scher et al., 2005; Vetvik & MacGregor, 2017; Wang et al., 2010)

The analysis of the determinants of migraine is however characterized by several contrasting results, and it is affected by endogeneity and reverse causality problems. As an example, low-income people are more likely to have migraine, but several authors (e.g. Agosti, 2018; Bigal et al., 2004) provided evidence that migraineurs may have lower incomes because migraine interferes with educational and occupational function, causing a loss of income or the ability to rise from a low-income group. Sauro and Becker (2009), Cesur et al. (2015) and Kristoffersen et al. (2018) report that stress is one of the main factors affecting migraine. However, stress may affect people with low as well as high income and socioeconomic status, making the relationship between the last variables and migraine undetermined. As a matter of fact, the results of a large survey used in this paper report that migraine affects even to a greater extent some high-income people than low-income people. This evidence calls for the search for other common determinants affecting both types of people.

In this paper, we provide evidence on a new determinant of migraine related to socio-psychological factors: income dissatisfaction (ID). Indeed, we show that ID plays a more relevant role in migraines than realized income. People dissatisfied with their income belong to low as well as high income and socioeconomic status, likely for different reasons. High-income people are more likely to be dissatisfied with their income due to fierce competition in the marketplace and the necessity to outperform rivals (Gilbert et al., 2009; Ntoumanis & Biddle, 1998); by contrast, low-income people may be dissatisfied with their income because it is insufficient to meet their basic needs. Whatever the reasons, we posit that exposure to prolonged or permanent ID leads to stress, and it may trigger a migraine. Among the few works that address the relationship between stress and ID, Marotz-Baden (1988) shows that economic satisfaction is negatively correlated with general stress, and the latter is higher at a lower income. On the other hand, recent evidence based on a survey of 1,000 LinkedIn members currently employed in the US, shows that among people who earn an income of $200,000 or more, nearly 70% said they feel stressed.

We use a database with about 80,000 individual-level observations that are representative of the Italian population provided by the Italian Statistical Institute (ISTAT) to test the hypothesis that subjects who were more dissatisfied with their income suffer more from migraine. The crucial advantage of the ISTAT survey is that it asks to what extent individuals are satisfied with their income. In addition, the multipurpose ISTAT survey facilitates our assessment of the extent of migraines in the population, its socioeconomic determinants, comorbidities, and the correlation of ID with a proxy of stress (anxiety). First, we provide empirical evidence that ID is above average among categories with low as well as high realized income. Next, we use an instrumental variable probit model to estimate the effect of ID on migraines.

The main result of the paper is that ID increases migraines, and this relationship holds for all types of jobs included in the ISTAT survey, which include low-income as well as high-income people. In addition, the relationship is robust to realized income, other illnesses, and socioeconomic conditions. We also explore the potential interaction between anxiety and ID in predicting migraine. Under the assumption that ID generates stress, we find that anxiety – a proxy for stress (Bystritsky & Kronemyer, 2014; Lenzo et al., 2021; Zhang et al., 2020) – is a common determinant of both ID and migraines, and we also show that the marginal effect of anxiety on ID and migraines is higher for people more affected by migraines (women, married people, and people living in the Southern regions).

The ID hypothesis adds a new explanation to the literature on the determinants of migraines relative to the socioeconomic status and psychological factors (see Kristoffersen et al., 2018; Peterlin & Scher, 2013; Sauro & Becker, 2009; Stewart et al., 2013), and it is an example of the complex relationship between physical and mental health as pointed out by studies such as Antonaci et al. (2011), Ohrnberger et al. (2017) and Seng et al. (2017).

Among others, the ID hypothesis provides an explanation of contrasting evidence in the literature which indicates that migraine may occur for people with low as well as high income. Indeed, we show that dissatisfaction on realized income is the most important determinant of migraines. Finally, our evidence is based on a sample that is representative of the Italian population, and thus it does not suffer from sample selection biases.

In the next section, we describe the data and the methodology; Section 3 presents the results, and Section 4 discusses them. Section 5 concludes the paper.

## 2. Data and methodology

We use a database with about 80,000 individual-level observations that are representative of the Italian population.^1^ The information on the health conditions and use of health services in Italy is retrieved from the survey provided by the Italian Statistical Institute (ISTAT), entitled ‘Indagine Multiscopo sulle Famiglie Condizioni di salute e ricorso ai servizi sanitari,’ which is the largest and most unbiased of its kind. It collects detailed information on conditions in terms of health and quality of life and provides a wide range of information on the spread of chronic diseases, perceived health, disability conditions, lifestyles and prevention, and the use of health services. We use the most recent available wave of the survey which ran from September 2012 to June 2013. In this cross-sectional analysis of baseline data, respondents were between 15 and 90 years of age, and migraine is related to people who self-reported in the interview headache more than 50% days in a month. Analyses were stratified by gender (women = 36,841, men = 43,249).

Table A1 presents the description and the summary statistics of the variables used in the econometric analysis. While, Table A2 shows the correlation matrix and in Table A3 we report the Variance inflation factor (VIF). Given the values greater than 1, it indicates that there is no correlation between a given explanatory variable and any other explanatory variables in the model Table 1.

**Table 1.** Mean values of the variables used in the estimations.

Variable	Migraineurs	No-Migraineurs	Test of the difference between means *P-Value*
INCOME_DISSAT (1–4)	2.48	2.39	*0*.*0000*
AGE (IN YEARS)	51.82	52.32	*0*.*0067*
FEMALE	64.65%	42.46%	*0*.*0000*
HIGHEDU	12.80%	13.28%	*0*.*1834*
MARRIED	59.31%	58.28%	*0*.*0499*
SOUTH	36.79%	34.57%	*0*.*0000*
ITACIT	95.86%	94.05%	*0*.*0000*
ASTHMA	8.28%	3.99%	*0*.*0000*
ALLERGY	22.14%	10.20%	*0*.*0000*
CELIAC	0.89%	0.38%	*0*.*0000*
HEART_PRO	7.13%	4.49%	*0*.*0000*
STROKE	2.26%	1.56%	*0*.*0000*
BULIMIA	1.42%	0.33%	*0*.*0000*
THYROID	11.63%	5.12%	*0*.*0000*
DEPRESSION	13.98%	3.58%	*0*.*0000*
ANXIETY	10.02%	2.38%	*0*.*0000*

For the description of variables see Table A1. H0: Equal mean among groups.

Table A1 in appendix shows that on average around 14% of Italians (19.3% women and 8.8% men) suffer from migraines which is higher than the worldwide average of migraines (around 11% according to the 2019 Global Burden of Disease Study). The most frequent occurrence of migraines occurs in both men and women between 41 and 50 years old.

The nature of the dependent variable MIGRAINE – defined as a dichotomous – drives the methodology approach employed. Indeed, we estimate a probit model to properly fit the probability that observation with specific attributes will fall into a particular one of the categories (Greene, 2003). Besides, reverse causality may arise between the probability of suffering from migraine and income dissatisfaction. Indeed, not only higher income dissatisfaction might influence migraine, but the latter may affect income dissatisfaction through a psychological channel. Furthermore, migraine and income dissatisfaction could be simultaneously determined by other potential confounding variables (i.e. individual characteristics), thus facing potential omitted variable problems. Hence, to control for endogeneity problems, we rely on an instrumental variable probit model.

Specifically, to test our research hypothesis, we fit several variants of the following instrumental variables probit model:

$$MIGRAINE={\text{β}}_{0}+{\text{β}}_{1}INCOME\_\;DISSAT_{i}+\phi X_{i}+\epsilon$$

where for each individual *i,* MIGRAINE is equal to one if an individual suffers from a migraine, and zero otherwise. INCOME_DISSAT is our main regressor, indicating the level of income dissatisfaction.^2^ Following several works in the literature (see, among others, Cesur et al., 2015; Ohrnberger et al., 2017; Stewart et al., 1991, 1992, 2013) we add a vector X of control variables that include: the age of the individual and its square (AGE and AGE2);^3^ a dummy equal to one if a subject is female (FEMALE); and a set of dummy variables accounting for whether an individual has Italian citizenship (ITACIT), she/he is married (MARRIED) or lives in the southern Italian regions (SOUTH), the average income level of the category (INCOME);^4^ and a dummy variable to account for employment/unemployment condition (EMPLOYED).

Specifically, we estimate three models, in which we add various fixed effects that could capture any individual-specific effect that we cannot control for:

Model 1 in which we add to the main model dummy variables to control for the sector in which the individuals work:^5^

$$MIGRAINE={\text{β}}_{0}+{\text{β}}_{1}INCOME\_DISSAT_{i}+\phi X_{i}++\sum_{s}\gamma_{s}\;SECTOR_{s}+\epsilon$$

Model 2, adding the type of job dummies:

$$MIGRAINE={\text{β}}_{0}+{\text{β}}_{1}INCOME\_DISSAT_{i}+\phi X_{i}+\sum_{p}\omega_{p}\;JOB_{p}+\epsilon$$

Model 3, adding region dummies capturing the area of provenience of the individual:

$$MIGRAINE={\text{β}}_{0}+{\text{β}}_{1}INCOME\_DISSAT_{i}+\phi X_{i}+\sum_{r}\varphi_{r}\;REGION_{r}+\epsilon$$

As a further robustness check, we add to the models 1, 2 and 3 a set of binary variables that account for individuals’ comorbidities.^6^ As additional evidence, we test whether the general results between ID and migraines hold when we estimate the baseline equation for each category.

We use instrumental variable estimations. As an external instrument, we use a dummy if the person has a higher education level (HIGHEDU), indeed, it has a correlation of −0.0071 with migraine and 0.1503 with income dissatisfaction (see Table A2 in the Appendix). The reason behind using this variable is that the accumulation of knowledge through education is the main way for individuals to improve their human capital; and this human capital enables them to increase their income. Furthermore, high income can allow consumers to meet their individual preferences and needs better, and improve their level of utility, thus, providing them with a higher level of happiness as individuals (Blanchflower & Oswald, 2004). Indeed, Nikolaev (2018) showed that the higher the level of education of individuals, the more satisfied they were with most areas of life (finance, employment opportunities, neighborhood, local community, children at home), and Yang et al. (2022) provides evidence that the higher the education level of an individual, the higher his/her income level and the stronger his/her sense of happiness. In addition, Salinas-Jiménez et al. (2011) use data from the World Values Survey, and find that education shows a significant effect on life satisfaction independent of its effect on income, thus identifying a consumption component of education. On the other hand, Sabia and Rees (2011) show that migraine is related to individual heterogeneity in the form of personality not to educational attainment. Gitto et al. (2015) use high education as an instrumental variable in the analysis of depressed mood in Korea.

## 3. Estimations results

Table 2 reports the results of the estimations. Firstly, it is important to notice that the instrument used passes standard tests for weak instruments (the value of χ2 is well above the standard thresholds). In the first three columns, we control respectively for the sector, job position and regional fixed effects. In the last three columns, we control for comorbidities with other illnesses. According to the Akaike Information Criterion (hereafter AIC), reported at the bottom of Table 2, the models with individuals’ comorbidities variables fit the data better. Yet, when we compare the models with fixed effects, it appears that those with regional fixed effect is more appropriate. Table 2 shows that a unit increment of ID increases by about 0.1 the probability of developing a migraine (columns 1 and 2). This result is robust also when we control for other illnesses.^7^

**Table 2.** The impact of income dissatisfaction on migraine.

	1	2	3	4	5	6
INCOME_DISSAT	0.0970***	0.1086***	0.0745***	0.0945***	0.1041***	0.0690***
	0.0231	0.0345	0.0165	0.0238	0.0358	0.0171
AGE	0.0043***	0.0041***	0.0045***	0.0037***	0.0036***	0.0040***
	0.0005	0.0005	0.0005	0.0005	0.0006	0.0005
AGE2	−0.0000***	−0.0000***	−0.0000***	−0.0000***	−0.0000***	−0.0000***
	0.0000	0.0000	0.0000	0.0000	0.0000	0.0000
FEMALE	0.1087***	0.1105***	0.1091***	0.0816***	0.0840***	0.0823***
	0.0028	0.0027	0.0026	0.0029	0.0029	0.0028
MARRIED	0.0138***	0.0148***	0.0115***	0.0186***	0.0193***	0.0164***
	0.0035	0.0042	0.0031	0.0034	0.0040	0.0031
SOUTH	0.0000	0.0003	0.0348***	−0.004	−0.0036	0.0259***
	0.0045	0.0058	0.0079	0.0046	0.0060	0.0080
ITACIT	0.0778***	0.0772***	0.0696***	0.0631***	0.0625***	0.0546***
	0.0088	0.0102	0.0078	0.0091	0.0107	0.0080
INCOME	0.0000	0.0000	0.0000	0.0000	0.0000	0.0000
	0.0000	0.0000	0.0000	0.0000	0.0000	0.0000
EMPLOYED	0.0249***	0.0281***	0.0213***	0.0356***	0.0382***	0.0312***
	0.0061	0.0078	0.0052	0.0061	0.0078	0.0052
ASTHMA				0.0509***	0.0508***	0.0523***
				0.0066	0.0068	0.0065
ALLERGY				0.0940***	0.0934***	0.0940***
				0.0042	0.0043	0.0042
CELIAC				0.0347*	0.0349*	0.0362*
				0.0200	0.0200	0.0198
HEART_PRO				0.0345***	0.0342***	0.0356***
				0.0065	0.0068	0.0064
ICTUS				0.0173	0.0173	0.0198*
				0.0106	0.0107	0.0104
BULIMIA				0.1284***	0.1275***	0.1346***
				0.0207	0.0210	0.0204
THYROID				0.0654***	0.0651***	0.0648***
				0.0056	0.0057	0.0056
DEPRESSION				0.1424***	0.1412***	0.1468***
				0.0083	0.0093	0.0077
ANXIETY				0.1324***	0.1319***	0.1352***
				0.0091	0.0096	0.0088
Observations	72,520	72,520	72,520	63,489	63,489	63,489
R-2	0.0173	0.0121	0.0264	0.0487	0.0435	0.0607
AIC	48964.23	49340.87	48300.51	35338.8	35678.42	34540.55
First stage chi2	606.22	276.65	1172.19	518.36	233.57	988.25
First stage chi2 (*p*)	0.0000	0.0000	0.0000	0.0000	0.0000	0.0000
Fixed Effect	Sector	Job Position	Region	Sector	Job Position	Region

For the description of variables see Table A1. The dependent variable is MIGRAINE. Superscripts ***, ** and * denote statistical significance at the 1, 5 and 10 percent level, respectively. The standard errors (in italics) are robust to heteroskedasticity and autocorrelation.

Focusing on the other control variables, the probability of having migraines increases with age, up to about 54.45 years old.^8^ It is higher for women and married people, for people living in Southern regions, and for those with Italian citizenship relative to foreigners. Moreover, being employed increases the probability of being affected by a migraine. Finally, all comorbidity factors are significant in determining migraines, but depression, anxiety and bulimia are by far the most correlated illnesses related to migraines. These results are consistent with the literature (see; Agosti, 2018; Antonaci et al., 2011; Buse et al., 2019; Cesur et al., 2015; Vetvik & MacGregor, 2017). For example, Ferrante et al. (2012), Allena et al. (2015) and ISS (2018), among others, using the results of questionnaires on Italian people, find that the prevalence of headaches was higher in females than in males. In addition, the results are robust to different specifications of our model, and the magnitude of the effects does not vary noticeably. Interestingly, when we have ID among the regressors, realized income is not a significant determinant of migraine (see Table 2), in all our specifications of the model.

To further corroborate our findings, we estimate the equation for each job position using an IV probit model. ID has a positive influence on the probability of being affected by a migraine for those employed in the following job position: management, self-employed workers, employee and freelance (see Table 3). Furthermore, the largest effect of ID is on those employed in management, which supports the view that ID is a more relevant determinant of migraines than the level of income.

**Table 3.** The impact of income dissatisfaction on migraine by job position.

	1	2	3	4	5	6	7	8	9	10	11
INCOME_DISSAT	−0.0658	0.4743**	0.1060**	−0.0584	0.1522	0.2712	−0.0434	0.1387*	0.2471***	0.2641	0.0524
	0.1078	0.2391	0.0501	0.1415	0.2172	0.3852	0.2104	0.0734	0.0857	0.3226	0.3650
AGE	0.0045	0.0080**	0.0062***	0.0057***	0.0077	0.0062	0	0.0057**	−0.0008	−0.0002	0.0021
	0.0039	0.0034	0.0010	0.0016	0.0060	0.0135	0.0036	0.0028	0.0016	0.0125	0.0031
AGE2	−0.0001	−0.0001*	−0.0001***	−0.0001***	−0.0001	0.0000	0.0000	−0.0001*	0.0000	0.0000	0.0000
	0.0000	0.0000	0.0000	0.0000	0.0001	0.0002	0.0000	0.0000	0.0000	0.0001	0.0000
FEMALE	0.0615***	0.1029***	0.1098***	0.1107***	0.0634***	0.2358***	0.0915***	0.0998***	0.1040***	0.2262***	0.0782***
	0.0203	0.0160	0.0050	0.0053	0.0239	0.0835	0.0216	0.0148	0.0085	0.0470	0.0214
MARRIED	−0.0537***	0.0213	0.0214***	−0.001	0.0495	0.0081	−0.0052	0.009	0.0179*	0.0367	0.0335
	0.0197	0.0166	0.0071	0.0149	0.0495	0.0779	0.0216	0.0134	0.0109	0.1063	0.0280
SOUTH	0.0021	−0.0321	−0.0175**	0.0414	−0.0895	0.0205	0.0434*	−0.0223*	−0.0111	0.0622	0.0691**
	0.0163	0.0199	0.0071	0.0296	0.0587	0.0831	0.0261	0.0123	0.0174	0.0462	0.0317
ITACIT	−0.0259	0.0834	0.0918***	0.027	0.094	0.0052	0.1160***	0.0045	0.1033***	0.2083*	0.0635
	0.0771	0.0594	0.0194	0.0390	0.0776	0.1723	0.0254	0.0502	0.0298	0.1250	0.0781
EMPLOYED	0.0227	0.0908***	0.0171	−0.0111	−0.01	0.2186	−0.005	0.0233	0.0698***	0.1269	0.0255
	0.0266	0.0335	0.0112	0.0304	0.0276	0.2806	0.0788	0.0278	0.0229	0.1017	0.0604
Observations	1,469	2,916	20,102	29,893	923	128	1,655	2,984	10,051	375	2,024
R-2	0.01	0.034	0.018	0.006	0.045	0.008	0.03	0.02	0.02	0.11	0.028
Sample	Manager	Management	Employee	Subordinate worker	Apprentice	Home worker on behalf of a company	Entrepreneur (with at least one employee)	Freelance	Self-employed worker	Member of a cooperative for the production of goods and services	Family assistant

For the description of variables see Table A1. The dependent variable is MIGRAINE. Superscripts ***, ** and * denote statistical significance at the 1, 5 and 10 percent level, respectively.The standard errors (in italics) are robust to heteroskedasticity and autocorrelation.

Summarizing the estimation results, we can conclude that, similar to previous findings, migraine has a non-linear relationship with age (Jensen & Stovner, 2008; The Woldeamanuel & Cowan, 2017; World Health Organization, 2016), it is higher among women (Bigal et al., 2004; Stang et al., 1992; Stewart et al., 1991, 1992; Vetvik & MacGregor, 2017), married and employed people. In addition, migraine is highly correlated with depression and anxiety (Agosti, 2018; Buse et al., 2019; Cesur et al., 2015; Korolainen et al., 2019; Scher et al., 2005; Vetvik & MacGregor, 2017; Wang et al., 2010). However, by contrast to previous evidence (Agosti, 2018; Bigal et al., 2004; Fernández-de-Las-Peñas et al., 2010; Molarius et al., 2008; Queiroz et al., 2009; Stewart et al., 2013; Winter et al., 2012), realized income is not a significant determinant of migraine. Indeed, we show that ID is a major determinant of migraines. Molarius et al. (2008) find that subjects who were dissatisfied with their work and subjects who were worried about losing their job suffered more often from headache disorders than those who were not.

## 4. Discussion

We proved that ID is a widespread phenomenon, and it has a heterogeneous effect according to the job position and any level of income. While it is more intuitive why people at low levels of income are likely to be unsatisfied with their income, less evident is income dissatisfaction among those earning high income. First, these people may be unhappy if their goals and expectations about life are above their current condition. Indeed, there is evidence that Italian people with higher education levels also have higher expected goals (Marotz-Baden, 1988), hence they are also more likely to find that their income expectations are not met (Clark & Oswald, 1996). In addition, Jongbloed (2018) focused on the impact of higher education on the happiness of Europeans and found that people with higher levels of education are less likely to perceive a sense of accomplishment from their work. Another reason why people with high incomes may be dissatisfied is related to the rule of the game. In a market economy, people like managers and entrepreneurs may feel dissatisfied with their income, because they are subject to the necessity to outperform rivals (Gilbert et al., 2009; Ntoumanis & Biddle, 1998). In a market economy what is relevant is relative, not absolute performance, and this may generate ID and stress. Indeed, our results support this view, since ID has a bigger impact on migraine for management and self-employed workers (see Table 3). However, the relationship between migraine and their determinants is complex (Rasmussen, 1995; Stewart et al., 2013). Even though we do not rule out that migraine can have detrimental effects on job conditions and ID, our instrumental variables estimations provide robust evidence that ID is a main determinant of migraine. We claim that the link between ID and migraine is stress: ID generates stress, and the latter is an important migraine trigger (Kaniecki, 2002).

As a matter of fact, according to the American Migraine Foundation, stress triggers migraines in almost 70% of sufferers. Stress is cited as the overall most common precipitating factor for migraines by Rasmussen (1993), Sauro and Becker (2009), Peroutka (2014), Cesur et al. (2015), Kristoffersen et al. (2018). On the other hand, there is evidence that stress may occur at low as well as high levels of income. Kahneman and Deaton (2010) show that emotional well-being rises at the beginning of low income, but there is no further progress beyond an annual income of ∼$75,000. Recent evidence that is based on a survey of 1,000 LinkedIn members currently employed in the US, shows that people who earn between $51,000 and $75,000 generally feel the least stressed. By contrast, of those who make an income of $200,000 or more, nearly 70% said they feel stressed.

Even though a direct measure of stress does not exist, the literature (Bystritsky & Kronemyer, 2014; Lenzo et al., 2021; Zhang et al., 2020) points out that anxiety can be considered a proxy for stress. Hence we estimate whether anxiety is a common determinant of ID and migraines. We estimated the correlation between anxiety, migraines, and ID. First, anxiety is more correlated to ID and migraine than other variables (see Table A2 in the appendix). In addition, anxiety is higher for people more affected by migraines (women, married people, and people living in the Southern regions). These results support previous findings (Capone et al., 2019; Petrillo et al., 2015), which report significantly higher levels of stress, sadness, and worry among Italian women than men. The reasons are that women have much greater economical and psychological barriers than men, and their unemployment rate is higher (Eurostat, 2017). In this paper, we take the view that ID can generate stress at any level of income, and we show that the higher the income dissatisfaction the higher the probability of having a migraine. In addition, there is evidence that stress can help initiate migraines in those predisposed to the disorder and may also contribute to chronic migraines (Sauro & Becker, 2009).

In addition to anxiety, the other important determinant of migraine is depression (Table 2), and the latter has also a significant correlation with ID (see Table A2). Apart from the direct effect of depression on migraine pointed out in the literature (Seng et al., 2017; Wacogne et al., 2003), we claim that depression has an indirect effect on migraine, due to the fact that depressed people are more likely to be dissatisfied with the life and also with their income. Indeed, in our sample depression has the highest correlation with ID relative to all the illnesses reported in Table A2.

However, we are aware that migraine is a complex phenomenon, and there are other determinants of migraine not included in the present work. To provide a few examples, caffeine and alcohol are among the most important triggers of migraines (see: https://americanmigrainefoundation.org), and they may relate also to ID. In addition, very recently Chu et al. (2021) established an association between insomnia and migraine risk. On the other hand, we provided evidence of the existence of a new determinant of migraine related to psychological factors, even though the relationship between psychological and other determinants triggering migraine remains an open issue.

Our work presents some limitations. First, the presence of omitted variables might be a serious concern as we might miss some covariates that could affect both the main regressors and the dependent variable. However, as we employ an instrumental variable approach, this may not be a relevant concern. What is more, the investigation is limited to one country, Italy, that has specific job-market characteristics, implying that more general findings require to test the models in other countries.

## 5. Conclusion

Using a large database representative of the Italian population, we tested the hypothesis that ID is a main determinant of migraine. The instrumental variables estimations show that ID increases migraine, and this result is robust to socio-economic conditions and comorbidities with other illnesses. Moreover, this relationship holds when we take account of realized income, and we estimate separately the equation for people in different job conditions. The ID hypothesis adds to the determinants of migraine, and it explains the contrasting evidence in the literature on the effects of realized in – come on migraine. In addition, it provides an explanation for why migraines may be above average among most developed (European countries) as well as least developed (Central and South America) countries.

Our results have important implications related to welfare and public policy. First, it shows that high inequality in the income distribution may have detrimental health effects not only for the poorer but also for the richer. In our approach migraine may be considered to some extent a negative externality generated by a market economy, which puts people at low as well as high income under too much stress. A policy implication of our results is that to contain migraine it is necessary to reduce the factors generating stress and income dissatisfaction. To this aim, the map of migraine is an important indicator of what sectors, jobs and geographical areas are more in need of welfare-improving policies. However, our results suggest that basic income welfare policies as well as business support policies have important health effects, that are necessary to take into account.

We tested the income dissatisfaction hypothesis with Italian data. Hence a natural extension of this research is to find additional evidence on other countries.

## Data Availability

The data that support the findings of this study are available from ISTAT (Italian national statistical institute) but restrictions apply to the availability of these data, which were used under license for the current study, and so are not publicly available.

## Notes

1 Exemption of the ethical approval has been granted from the ethical committee of the University of Calabria.

2 Precisely, the question in the survey is the following: ‘How do you consider the total economic resources of your family in the last 12 months’ and the possible answers are: ‘excellent, adequate, scarce, absolutely insufficient’. As a result, the variable ranges from 1 (excellent) to 4 (absolutely insufficient); hence, ID increases with higher values of the variable.

3 In Table A4 we report the results by replacing AGE and AGE2 with a categorial dummy for age. Specifically, we define the following categories: young, adult and mature.

4 Further, we do not have information about the individual income level. Thus, we use the average income level for each job position provided by the Italian Revenue Agency.

5 We consider the following sectors of activity: agriculture, forestry, and fishing; mining; manufacturing; construction; wholesale and retail trade; repair of cars and motorcycles; accommodation and restaurant services; transport and ware- housing; financial and insurance activities; real estate activities; public administration; defence; and compulsory social insurance; education; health and social assistance; arts, entertainment, and entertainment activities; and repair of house – hold goods and other services. Job position are top management; middle / lower management; employee; chief worker, subordinate worker and similar; apprentice; company employee working at home; entrepreneur (with at least one em- ployee); freelance; self-employed worker; member of a cooperative for the production of goods and services; and family assistant.

6 Comobidities are dummy variables capturing whether the individual suffer from other illnesses.

7 The evidence reported in Table 2 is confirmed also by employing a standard logit model. For the sake of brevity, these results are not included but available upon request.

8 The main results are also confirmed when we replace AGE and AGE2 with a categorial dummy for age (Table A4 in the Appendix).

## Appendix

**Table A1.** Description and summary statistics of the variables used in the estimations.

VARIABLE	DESCRIPTION	Mean	StdD	Min	Max	Obs
INCOME_DISSAT	Level of economic dissatisfaction of the individual	2.40	0.62	1	4	80,091
MIGRAINE	Dummy equal to one if the individual is affected by migraine, zero otherwise	0.14	0.34	0	1	74,921
AGE	Age of the individual	52.50	17.16	15	90	80,091
FEMALE	Dummy equal to one if the individual is a female, zero otherwise	0.463	0.499	0	1	80,091
HIGHEDU	Dummy equal to one if the individual is higher educated, zero otherwise. See table notes.	0.132	0.338	0	1	80,091
MARRIED	Dummy equal to one if the individual is married, zero otherwise	0.58	0.49	0	1	80,091
SOUTH	Dummy equal to one if the individual is resident in the southern part of Italy, zero otherwise	0.35	0.48	0	1	80,091
ITACIT	Dummy equal to one if the individual is an Italian citizen, zero otherwise	0.94	0.23	0	1	80,091
INCOME	Hour wage per job position in euro	31.44	99.85	9.45	723.00	77,499
EMPLOYED	Dummy equal to one if the individual is employed, zero otherwise	0.53	0.50	0	1	80,091
ASTHMA	Dummy equal to one if the individual is affected by asthma, zero otherwise	0.05	0.21	0	1	77,707
ALLERGY	Dummy equal to one if the individual is affected by allergy, zero otherwise	0.12	0.32	0	1	76,810
CELIAC	Dummy equal to one if the individual is celiac, zero otherwise	0.00	0.07	0	1	80,000
HEART_PRO	Dummy equal to one if the individual is affected by heart problem, zero otherwise	0.05	0.22	0	1	79,169
STROKE	Dummy equal to one if the individual had a stroke, zero otherwise	0.02	0.13	0	1	80,091
BULIMIA	Dummy equal to one if the individual is affected by bulimia, zero otherwise	0.00	0.07	0	1	79,588
THYROID	Dummy equal to one if the individual has thyroid problem, zero otherwise	0.06	0.24	0	1	78,789
DEPRESSION	Dummy equal to one if the individual is affected by depression, zero otherwise	0.05	0.22	0	1	76,656
ANXIETY	Dummy equal to one if the individual suffers of anxiety, zero otherwise	0.04	0.19	0	1	78,719

Authors’ elaboration on ISTAT data.

As higher educated people we include those with a Bachelor, Master or Doctorate.We do not consider Italian citizens as those with foreign citizenship. The economic dissatisfaction refers to income: the measure varies from 1 (fully satisfied) to 4 (no satisfied).

**Table A2.** Correlation matrix.

MIGRAINE	INCOME_DISSAT	AGE	FEMALE	HIGHEDU	MARRIED	SOUTH	ITACIT	INCOME	EMPLOYED	ASTHMA	ALLERGY	CELIAC	HEART_PRO	STOKE	BULIMIA	THYROID	DEPRESSION	ANXIETY	
MIGRAINE	1																		
INCOME_DISSAT	0.0451	1																	
AGE	−0.0065	−0.053	1																
FEMALE	0.1406	−0.0131	0.0011	1															
HIGHEDU	−0.0071	−0.1503	−0.1213	0.062	1														
MARRIED	0.0095	−0.069	0.194	−0.0801	−0.0419	1													
SOUTH	0.0116	0.1221	−0.0289	−0.0737	−0.0002	0.0426	1												
ITACIT	0.0253	−0.1248	0.1762	−0.0219	0.0168	0.0527	0.0959	1											
INCOME	−0.0189	−0.0962	0.0553	−0.06	0.2259	0.0364	−0.0088	0.0343	1										
EMPLOYED	0.0044	−0.1062	−0.5679	−0.0984	0.1686	−0.0504	−0.0346	−0.0886	0.0132	1									
ASTHMA	0.068	0.0316	0.0574	0.0048	−0.0179	0.0029	0.0038	0.0118	−0.0117	−0.0511	1								
ALLERGY	0.1173	0.01	−0.0631	0.0519	0.03	−0.019	−0.0222	0.0155	−0.0052	0.0356	0.2055	1							
CELIAC	0.0221	0.004	−0.0173	0.0351	0.009	−0.0051	−0.0023	0.0118	−0.0009	0.0058	0.0291	0.0403	1						
HEART_PRO	0.0375	0.0264	0.2238	−0.0137	−0.033	0.0065	0.0051	0.04	0.0127	−0.1626	0.0714	0.0167	0.0084	1					
STOKE	0.015	0.0193	0.1556	−0.016	−0.0347	0.0038	0.001	0.0217	−0.0073	−0.1163	0.0247	−0.0044	0.0099	0.0985	1				
BULIMIA	0.0562	0.0228	0.0072	0.0222	0.0031	−0.0074	−0.0061	0.004	0.0035	−0.006	0.0349	0.0287	0.0393	0.0233	0.0123	1			
THYROID	0.0888	0.0019	0.0817	0.1842	−0.0094	0.0193	−0.003	0.0289	−0.005	−0.0733	0.0409	0.0539	0.0217	0.0676	0.012	0.0313	1		
DEPRESSION	0.1529	0.0855	0.1437	0.067	−0.04	−0.0311	0.002	0.0298	−0.0162	−0.1418	0.0744	0.0413	0.0149	0.1068	0.096	0.1191	0.073	1	
ANXIETY	0.142	0.0712	0.1253	0.0671	−0.0352	−0.0251	0.0179	0.0324	−0.0098	−0.1172	0.0703	0.0494	0.0177	0.1003	0.0687	0.1185	0.0713	0.4946	1

For the description of the variables see Table A1.

**Table A3.** Variance inflation factor (VIF).

Variable	VIF
AGE	1.70
FEMALE	1.07
MARRIED	1.06
SOUTH	1.04
ITACIT	1.06
INCOME	1.02
EMPLOYED	1.56
ASTHMA	1.06
ALLERGY	1.06
CELIAC	1.01
HEART_PRO	1.07
STROKE	1.04
BULIMIA	1.02
THYROID	1.05
DEPRESSION	1.36
ANXIETY	1.35
Mean VIF	1.15

For the description of variables see Table A1.

**Table A4.** The impact of income dissatisfaction on migraine using age categories.

	1	2	3	4	5	6
INCOME_DISSAT	0.1023***	0.1162***	0.0749***	0.0968***	0.1086***	0.0673***
	0.0237	0.0357	0.0170	0.0245	0.0372	0.0177
**Baseline: Young**						
Adult	0.0401***	0.0417***	0.0424***	0.0281***	0.0300***	0.0299***
	0.0046	0.0047	0.0045	0.0046	0.0047	0.0045
Mature	0.0125*	0.0206**	0.0122*	−0.0002	0.0074	−0.0021
	0.0069	0.0089	0.0064	0.0076	0.0100	0.0068
FEMALE	0.1069***	0.1092***	0.1076***	0.0802***	0.0830***	0.0809***
	0.0028	0.0028	0.0027	0.0030	0.0030	0.0028
MARRIED	0.0163***	0.0173***	0.0138***	0.0211***	0.0219***	0.0189***
	0.0034	0.0040	0.0031	0.0033	0.0038	0.0030
SOUTH	−0.0019	−0.0018	0.0335***	−0.005	−0.005	0.0251***
	0.0045	0.0059	0.0079	0.0047	0.0062	0.0080
ITACIT	0.0826***	0.0826***	0.0736***	0.0658***	0.0659***	0.0566***
	0.0089	0.0105	0.0079	0.0092	0.0110	0.0081
INCOME	0.0000	0.0000*	0.0000	0.0000	0.0000	0.0000
	0.0000	0.0000	0.0000	0.0000	0.0000	0.0000
EMPLOYED	0.0158**	0.0192**	0.0104*	0.0293***	0.0320***	0.0233***
	0.0069	0.0091	0.0058	0.0069	0.0090	0.0058
ASTHMA				0.0066	0.0068	0.0065
				0.0939***	0.0931***	0.0940***
ALLERGY				0.0042	0.0043	0.0042
				0.0340*	0.0340*	0.0354*
CELIAC				0.02	0.02	0.0198
				0.0333***	0.0334***	0.0346***
HEART_PRO				0.0064	0.0066	0.0063
				0.0136	0.0146	0.0161
ICTUS				0.0105	0.0105	0.0104
				0.1278***	0.1268***	0.1346***
BULIMIA				0.0207	0.021	0.0204
				0.0670***	0.0667***	0.0666***
THYROID				0.0056	0.0057	0.0056
				0.1410***	0.1399***	0.1460***
DEPRESSION				0.0082	0.0092	0.0077
				0.1323***	0.1317***	0.1355***
ANXIETY				0.0091	0.0096	0.0088
				0.0091	0.0096	0.0088
Observations	72,520	72,520	72,520	63,489	63,489	63,489
R-2	0.0155	0.0087	0.0268	0.0477	0.0411	0.0616
Fixed Effect	Sector	Job Position	Region	Sector	Job Position	Region

For the description of variables see Table A1. The dependent variable is MIGRAINE. Superscripts ***, ** and * denote statistical significance at the 1, 5 and 10 percent level, respectively. The standard errors (in italics) are robust to heteroskedasticity and autocorrelation. Young refers to people from 15 to 30 years old. Adults refers refers to people from 31 to 60 years old, while the rest refers to mature category.
